# Nanoparticle-Based Secretory Granules Induce a Specific and Long-Lasting Immune Response through Prolonged Antigen Release

**DOI:** 10.3390/nano14050435

**Published:** 2024-02-27

**Authors:** Laia Bosch-Camós, Carlos Martínez-Torró, Hèctor López-Laguna, Jara Lascorz, Jordi Argilaguet, Antonio Villaverde, Fernando Rodríguez, Esther Vázquez

**Affiliations:** 1 Unitat Mixta d’Investigació IRTA-UAB en Sanitat Animal, Centre de Recerca en Sanitat Animal (CReSA), Campus de la Universitat Autònoma de Barcelona (UAB), 08193 Bellaterra, Spainjordi.argilaguet@irta.cat (J.A.); 2 Institut de Recerca i Tecnologia Agroalimentàries (IRTA), Programa de Sanitat Animal, Centre de Recerca en Sanitat Animal (CReSA), Campus de la Universitat Autònoma de Barcelona (UAB), 08193 Bellaterra, Spain; 3 WOAH Collaborating Centre for the Research and Control of Emerging and Re-Emerging Swine Diseases in Europe (IRTA-CReSA), 08193 Bellaterra, Spain; 4 CIBER de Bioingeniería, Biomateriales y Nanomedicina (CIBER-BBN, ISCIII), Universitat Autònoma de Barcelona, 08193 Bellaterra, Spain; carlosmartineztorro@gmail.com (C.M.-T.); hector.lopez@uab.cat (H.L.-L.); jara.lascorz@gmail.com (J.L.); esther.vazquez@uab.es (E.V.); 5 Institut de Biotecnologia i de Biomedicina, Universitat Autònoma de Barcelona, 08193 Bellaterra, Spain; 6 Departament de Genètica i de Microbiologia, Universitat Autònoma de Barcelona, 08193 Bellaterra, Spain

**Keywords:** recombinant proteins, biomaterials, protein nanoparticles, secretory granules, immunization

## Abstract

Developing prolonged antigen delivery systems that mimic long-term exposure to pathogens appears as a promising but still poorly explored approach to reach durable immunities. In this study, we have used a simple technology by which His-tagged proteins can be assembled, assisted by divalent cations, as supramolecular complexes with progressive complexity, namely protein-only nanoparticles and microparticles. Microparticles produced out of nanoparticles are biomimetics of secretory granules from the mammalian hormonal system. Upon subcutaneous administration, they slowly disintegrate, acting as an endocrine-like secretory system and rendering the building block nanoparticles progressively bioavailable. The performance of such materials, previously validated for drug delivery in oncology, has been tested here regarding the potential for time-prolonged antigen release. This has been completed by taking, as a building block, a nanostructured version of p30, a main structural immunogen from the African swine fever virus (ASFV). By challenging the system in both mice and pigs, we have observed unusually potent pro-inflammatory activity in porcine macrophages, and long-lasting humoral and cellular responses in vivo, which might overcome the need for an adjuvant. The robustness of both innate and adaptive responses tag, for the first time, these dynamic depot materials as a novel and valuable instrument with transversal applicability in immune stimulation and vaccinology.

## 1. Introduction

**Figure 1:**
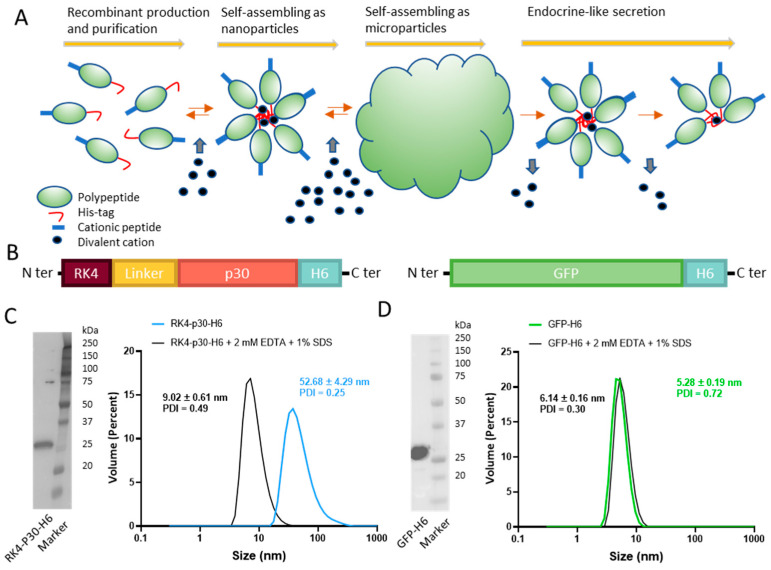
Construction and characterization of RK4-P30-H6 and GFP-H6. (**A**) Architectonic principles governing the POM principle, namely microparticle generation out of nanoparticles and further nanoparticle release. His-tagged proteins tend to self-assemble, upon recombinant production and Ni^2+^-based purification, into oligomeric nanoparticles, assisted by divalent cations. A cationic amino acid N-terminal stretch favors this process. The addition of a molar excess of cationic Zn produces the immediate formation of microscale particles. Upon in vivo administration and upon Zn dilution, these materials release stable nanoparticles differently. (**B**) Schematic representation of RK4-P30-H6 and GFP-H6 constructs. In RK4-P30-H6, a flexible peptide linker (GGSSRSS) was incorporated. (**C**) RK4-P30-H6 characterization by H6 immunodetection in Western blot with anti-His monoclonal antibody ((**C**), left). Size of the purified protein determined by DLS. The protein size was also measured under chelating conditions (2 mM EDTA + 1% SDS) ((**C**), right). (**D**) Immunodetection of GFP-H6 by Western Blot ((**D**), left) and size of the construct analyzed by DLS ((**D**), right).

Protein materials, that is, supramolecular protein complexes with defined physicochemical and biological properties, are gaining interest in biomedicine because of their potent applications in drug delivery and in regenerative medicine [1,2,3,4,5]. Several approaches allow the controlled oligomerization of selected polypeptides into fibrils, layers, matrices, nanoparticles, or microparticles [6,7,8,9]. One of the most versatile, promising and technologically simple strategies for protein assembly is the exploitation of the coordination capabilities between divalent cations (such as Zn^2+^, Ca^2+^, Mg^2+^ and Mn^2+^) and histidine (His) residues. This category of interactivity allows the cross-molecular binding of poly-His-tagged proteins into nanoparticles. When increasing the amounts of crosslinking ions, these materials are progressively clustered as granular microscale particles [10,11]. Both processes can be reversed by the exposure to chelating agents or, more slowly, by the mere physiological equilibrium-based dilution of the gluing cation (Figure 1A). This fact results in the progressive disintegration of the material into their forming building blocks, either nanoparticles or their protomers, that are released to the media [12]. In addition, it is known that the formation of the nanoparticle building blocks is favored if the N-terminal segment of the polypeptide is cationic [13]. Taking this approach and by regulating the cation/His ratio in the coordination mixture, both nanoparticles and microparticles have been generated in a controlled way through robust and highly efficient protocols that keep the folding status and functionality of the forming polypeptides. While the intermediate nanoparticles have been mostly adapted as vehicles for the cell-targeted delivery of small molecular weight drugs and cytotoxic protein in oncology [14,15], microparticles show appealing properties as slow drug delivery systems [16,17].

Once these micron-scale materials are administered subcutaneously, they leak the forming polypeptides that reach the bloodstream [17]. Notably, if fused to specific ligands of cell surface receptors, the released protein accumulates in receptor-overexpressing target organs [17]. This principle, which mimics the secretion process of peptide hormones in the mammalian endocrine system [18,19,20,21,22,23], has been explored for the delivery of diverse functional proteins with therapeutic applications [5]. A main advantage of this system over other slow drug delivery platforms is that the protein drug is self-contained as mechanically stable dynamic depot in the absence of any chemically heterogeneous scaffold. Thus, inert holding materials or matrices, that might pose compatibility issues, are not needed here to support the endocrine-like character of these protein materials.

The fact that these secretory microparticles undergo a disintegration process in vivo results in a time-prolonged release of functional, properly folded proteins, in contrast to a conventional single shot. The leakage profile is dependent on protein properties and on the used gluing cation [12]. This concept might be highly appealing in vaccinology as a way to expose a given antigen to the immune system during a prolonged period upon a single administration, mimicking the immune stimulation during a natural infection. Therefore, secretory microparticles might represent a novel approach to clinical immunization based on the subcutaneous implantation of antigen-delivering protein materials. This possibility has been explored here through the preparation and immunogenicity evaluation of protein-only secretory materials, utilizing two distinct antigens and in two different experimental models. Firstly, the in vivo safety and immunogenicity of protein-only microparticles based on p30 (also named p32) [24], a main antigen of the African swine fever virus (ASFV) [25,26], were examined in pigs. This structurally complex virus [25] causes severe hemorrhagic disease in domestic pigs [27,28]. Being a significant global veterinary concern [26,29,30,31,32], primarily due to the lack of effective vaccine and vaccine prototypes [33], it is particularly worrying in large countries such as China in which the pig industry is a strong economic supporter [34,35]. Secondly, a mouse model was employed to assess the safety and immunogenicity of secretory granules based on the green fluorescent protein (GFP). Additionally, to obtain a deeper understanding of the immunological mechanisms triggered by these materials, the specific cytokine profile induced in vitro in primary cells was examined. The obtained data have been evaluated in the context of simple, cost-effective, and efficient new generation protein-based vaccination platforms that, based on sustained antigen release, might offer appealing properties over the current immunization methods.

## 2. Materials and Methods

### 2.1. Ethics Statement

Animal care and procedures were performed in accordance with the guidelines of the Good Experimental Practice and with the approval of the Ethics Committee on Animal Experimentation of the Generalitat de Catalunya (pig experiments project codes: CEA-OH/11580/1 and CEA-OH/10298/2; mice experiment project code: CEA-OH/11691/2).

### 2.2. Protein Design, Production and Purification

The in-house designed synthetic gene for the modular protein RK4-P30-H6 was provided by Gene Art (Thermo Fisher, Waltham, MA, USA), and subcloned in a pET22b plasmid (Novagen, Madison, WI, USA). The construct contains four repetitions of RK at the N-terminal that serve as a cationic peptide to stimulate nanoparticle formation together with the poly-His tag at the C-terminal end. The production of the protein was tested at 20 °C (overnight) and 37 °C (3 h) with two different concentrations of IPTG (0.1 mM, 1 mM) in an *Escherichia coli* BL21 strain. Protein production was optimized at 20 °C overnight with 0.1 mM IPTG. After the production, cells were harvested at 5000× *g* for 15 min and the pellets were washed with PBS and stored at −80 °C until further use. Prior to the purification, cells were resuspended in Wash Buffer (20 mM Tris-HCl pH 8, 500 mM NaCl and 10 mM imidazole) with a cOmplete Protease Inhibitor Cocktail EDTA-free tablet (Roche Diagnostics, Rotkreuz, Switzerland). Cells were then disrupted in an EmulsiFlex-C5 system (Avestin, Ottawa, ON, Canada) for 3 rounds at approximately 7500 psi. After the disruption, the mixture was centrifuged at 15,000× *g* for 45 min and the soluble fraction was retained and filtered through sterile 0.22 µm filters (Millipore, Burlington, MA, USA). Next, the filtered soluble fraction was loaded into a HisTrap HP 5 mL column (GE Healthcare, Chicago, IL, USA) using an ÄKTA Pure chromatography system (GE Healthcare). Elution was performed through a linear gradient of Elution Buffer (20 mM Tris-HCl pH 8, 500 mM NaCl, 500 mM imidazole). The fractions were analyzed by SDS-PAGE and Western blot (anti-His tag, Genscript, Piscataway, NJ, USA) and those containing RK-P30-H6 were dialyzed thrice against a saline buffer (166 mM NaCO_3_H, 333 mM NaCl).

### 2.3. Dynamic Light Scattering

The volume size distribution of nanoparticles was determined by Dynamic Light Scattering (DLS) at 633 nm (Zetasizer Pro, Malvern, Malvern, UK). Samples were diluted in their respective buffer to a concentration of 1 mg/mL. The samples were measured in triplicate.

### 2.4. Microparticle Formation and Protein Release

RK4-p30-H6 and GFP-H6 (at 1 mg/mL) were mixed individually with a zinc chloride solution at a 200:1 divalent cation-to-protein ratio. The mixture was incubated at room temperature for 10 min, and then the microparticles were recovered by centrifugation (10,000× *g*, 5 min). The supernatant was discarded. To assess the release of protein from these microparticles, these materials were resuspended in PBS (diluted to a final concentration of 1 mg/mL) and further incubated at 37 °C for seven days. Samples were extracted on day 0, 1, 3, and 7. Then, we loaded the samples into a polyacrylamide gel which was later transferred to a PVDF membrane. The percentage of protein released during the experiment was assessed by Western Blot using an anti-polyhistidine tag antibody. Images were processed with the Image Lab (BioRad, Hercules, CA, USA) software (v. 6.1).

### 2.5. Scanning Electron Microscopy

High-resolution images of cation-induced microparticles were obtained by field emission scanning electron microscopy (FESEM). A volume of 10 μL of each microparticle sample (0.5 mg/mL) was deposited on silicon wafers (Ted Pella Inc., Redding, CA, USA) overnight and then observed, without coating, in a FESEM Zeiss Merlin (Zeiss, Jena, Germany) operating at 1 kV and equipped with a high-resolution secondary electron detector.

### 2.6. Study Design of the Pig Experiments

Landrace × Large White male piglets at 8 weeks of age at arrival were used. Pigs were housed in the experimental farm of IRTA Monells (Girona, Spain). Animals were fed ad libitum, and an acclimation period of one week was allowed before the initiation of the study. In the first experiment, animals were subcutaneously inoculated once or twice three weeks apart with 50 µg (final volume of 0.5 mL) of the soluble or protein-only-microparticles (POMs) form of the ASFV protein p30 in sodium carbonate buffer (166 mM NaHCO_3_, 333 mM NaCl buffer, pH 8), in the presence or absence of CAF01 (half of the volume, following manufacturer’s instructions). A group of three pigs received PBS following the same procedure as controls. Blood samples were collected weekly. In the second experiment, pigs received either a high dose of 150 µg of p30 POMs or PBS (control group), following the same immunization regimen as experiment 1.

### 2.7. Study Design of the Mice Experiment

Sixteen BALB/c mice at 7 weeks of age at arrival were used (half females and half males). Mice were allocated to cages according to sex and fed ad libitum during the experiment. Ear cuts were used to differentiate the animals. After one week of acclimation, six mice (3 females and 3 males) were subcutaneously inoculated twice 3 weeks apart with 50 µg of GFP POMs, while six others (3 females and 3 males) received 5 µg of the same antigen following the same regimen. GFP POMs were suspended in sodium carbonate buffer (166 mM NaHCO_3_, 333 mM NaCl buffer, pH 8), and the inoculated volume was 0.3 mL. Four mice (2 females and 2 males) were used as controls and received 0.3 mL of PBS in each administration. Blood for the generation of sera was taken from the facial vein before the first inoculation (SD0), and 2 and 9 weeks after the second inoculation (SD35 and SD85, respectively). Two weeks after the second immunization, three mice receiving a 50 µg/dose of GFP POMs, three receiving a 5 µg/dose, and two controls were euthanized to assess the cellular response induced by GFP POMs using splenocytes. The six remaining animals were kept for seven more weeks to analyze long-term immunogenicity.

### 2.8. ELISA

ASFV-specific antibodies were assessed by indirect ELISA based on the soluble extracts of ASFV-infected cells approved by the WOAH [36]. Sera were tested at a 1/100 dilution unless otherwise specified. For porcine IgG and IgA, peroxidase-conjugated rabbit anti-pig IgG (Sigma-Aldrich, A5670, St. Louis, MO, USA) at 1/20,000 or peroxidase-conjugated goat anti pig-IgA (Invitrogen, PA1-84625, Waltham, MA, USA) at 1/2500 were used. For specific isotypes, mouse anti-pig IgG1 (BioRad, MCA635GA) at 1/1000, mouse anti-pig IgG2 (BioRad, MCA636GA) at 1/1000, followed by peroxidase-conjugated goat anti-mouse polyvalent immunoglobulins (Sigma-Aldrich, A4012) at 1/2500 were used. In the case of GFP-specific antibodies, COSTAR 3590 Corning High binding 96-well plates (Cultek, 153590, San Fernando de Henares, Spain) were coated with the soluble form of the protein at 5 µg/mL in carbonate-bicarbonate buffer overnight at 4 °C. Plates were washed 3× with PBS-Tween20 0.05% (PBS-T), and blocked for 1 h at 37 °C with 5% CO_2_ with PBS-T 2% BSA. Afterwards, mouse serum samples in PBS-T 2% BSA were added and incubated for 1 h at 37 °C in 5% CO_2_. After incubation with the tested mice serum, one of the corresponding antibodies in PBS-T 2% BSA were used: HRP-conjugated goat anti-mouse polyvalent immunoglobulins (Sigma-Aldrich, A4012) at 1/1000, HRP anti-mouse IgG1 (Abcam, ab97240, Cambridge, UK) at 1/1000, HRP anti-mouse IgG2a heavy chain (Abcam, ab97245) at 1/1000, or HRP anti-mouse IgA alpha chain (Abcam, ab97235) at 1/1000. In both cases, soluble 3,3′,5,5′-tetramethylbenzidine (TMB, Sigma-Aldrich, T4444) was used as the specific chromogenic substrate for the HRP, and the reaction was stopped with 1 N H_2_SO_4_. Plates were finally read at a wavelength of 450 nm, and the average absorbance [optical density (OD) values] of duplicates are represented in the graphs.

### 2.9. Mouse Splenocytes Collection and Flow Cytometry

Mice splenocytes were obtained by the mechanical dissociation of spleens and filtration through a 40 µm cell strainer. Red blood cells were then lysed using NH4Cl for 5 min at room temperature, and splenocytes were finally suspended in RPMI 1640 medium (Gibco, Waltham, MA, USA) supplemented with 10% FBS (Cultek), 2 mM L-glutamine (Invitrogen), 100 IU/mL penicillin/streptomycin (Invitrogen), 0.05 mM β-mercaptoethanol, and 1 mM sodium pyruvate. Fresh mouse splenocytes were used for flow cytometry analysis. One million cells were used per condition in U-bottom 96-well plates (100 µL/well). Splenocytes were stimulated for 5 days with 5 µg/mL of GFP POMs. Complete RPMI was used as the negative control, while stimulation with phorbol myristate acetate (PMA) plus ionomycin (at 5 ng/mL and 500 ng/mL, respectively) was used as the positive control. After stimulation, cells were stained with the Zombie NIR fixable viability kit (Biolegend, 423106, San Diego, CA, USA) following manufacturer’s instructions, and then a blockage of Fc receptors was performed with PBS 5% FBS for 15 min on ice. Extracellular staining was performed for 20 min on ice in PBS 2% FBS using 50 µL of a mix of the following antibodies: APC hamster anti-mouse CD3e at a 1/20 dilution (BD Biosciences, #553066, Franklin Lakes, NJ, USA), PerCP-Cy5.5 rat anti-mouse CD4 at a 1/300 dilution (BD Biosciences, #550954), and PE-Cy7 rat anti-mouse CD8a at a 1/150 dilution (BD Biosciences, #552877). Afterwards, the BD Cytofix/Cytoperm Kit (BD Biosciences) was used according to the manufacturer’s protocol to fix and permeabilize the cells. Intracellular staining using BV421 mouse anti-Ki67 at a 1/150 dilution (BD Biosciences #652411) was then performed for 30 min on ice in Perm/Wash buffer (BD Biosciences). Samples were acquired on a BD FACSAria IIu flow cytometer (BD Biosciences) and data were analyzed using FlowJo v10.7.1 software (Tree Star Inc., San Carlos, CA, USA).

### 2.10. ELISpot Assay with Porcine Peripheral Blood Monocyte Cells (PBMCs)

PBMCs were separated from whole blood by density-gradient centrifugation with Histopaque 1077 (Sigma). Red blood cells from PBMC were lysed for 5 min with ammonium chloride. Final cell cultures were suspended in RPMI 1640 medium (Gibco) supplemented with 10% FCS, 100 IU of penicillin/streptomycin/mL (Invitrogen), 2 mM L-glutamine (Invitrogen), and 0.05 mM 2-mercaptoethanol. Trypan blue was used to assess cell viability. IFNγ-secreting cells were assessed by ELISpot assay using purified mouse anti-pig IFNγ (clone P2G10, BD Pharmingen) as the capture antibody and biotinylated mouse anti-porcine IFNγ antibody (clone P2C11, BD Pharmingen) as the detection antibody, following a previously reported method [36]. Cells were stimulated with p30 or p30 POMs at 5 µg/mL, and incubated for 16 h at 37 °C in 5% CO_2_.

### 2.11. Isolation of Porcine Alveolar Macrophages (PAMs)

PAMs were isolated from pig lungs following an adapted protocol [37]. Briefly, pigs were euthanized by exsanguination. The trachea was ligated to prevent total pulmonary collapse, followed by the removal of the heart and lungs from the thorax. Alveolar macrophages were collected in PBS from lungs by bronchioalveolar lavage. PAMs were cultured in complete RPMI-1640 medium [10% fetal bovine serum (FBS), 2 mM L-glutamine, 1 µg/mL fungizone, 100 U/mL penicillin, and 100 µg/mL streptomycin] in Petri dishes for 2 h at 37 °C in a humidified 5% CO_2_ atmosphere. Some PAMs were cultured for 5 days at 37 °C, and qPCR was performed to ensure the cells were negative for the presence of Porcine circovirus 2, Porcine reproductive and respiratory virus, and Mycoplasma. The remaining cells were stored in liquid nitrogen until use.

### 2.12. Multiplex Luminex Assay

PAMs were seeded in 24-well plates (10^6^ cells/well) in RPMI 1640 medium supplemented with 10% FBS, 100 IU/mL of penicillin/streptomycin, 2 mM L-glutamine, and 0.5% nystatin (sigma-MERCK) and incubated overnight at 37 °C in 5% CO_2_. Afterwards, the medium was removed and replenished with fresh RPMI either with or without 5 µg/mL of GFP POMs. After a 24 h incubation at 37 °C, 5% CO_2_, cell supernatants were collected and cytokines were determined using the Luminex xMAP technology and the ProcartaPlex Porcine Cytokine & Chemokine Panel 1 (Thermo Fisher Scientific). Cytokine concentrations were calculated using the xPONENT 4.3 software (Luminex, Austin, TX, USA) and expressed as pg/mL (except for TNF, for which a technical problem with the standard curve occurred and only the mean fluorescence intensity (MFI) values could be assessed).

### 2.13. Statistical Analyses

Prism version 8.3.0 software (GraphPad, La Jolla, CA, USA) was used to plot the results and perform statistical analyses. The tests used are specified on figure legends. Statistical significance was set at *p* < 0.05 and is displayed in GraphPad style (*p* > 0.05 ns, * *p* ≤ 0.05, ** *p* ≤ 0.01, *** *p* ≤ 0.001).

## 3. Results

### 3.1. Design and Construction of an ASFV-Antigen Nanoparticle

A full-length, proteolytically stable version of the antigen p30 [38], (also named p32) [24], was engineered and produced in *Escherichia coli* as a multidomain protein, placed between a cationic N-terminal peptide (RK4) and a C-terminal hexahistidine (H6) tail (RK4-P30-H6, Figure 1B). While this antigen by itself is not protective (as any other from the virus, [33]), it elicits potent specific antibody and cellular responses [39], representing a fully valid model for the testing of our concepts. In the recombinant construct, the combination of these end terminal peptides was expected to promote the self-assembling of the construct as oligomeric nanoparticles assisted by divalent cations from the media. A simpler GFP-H6 construct was used as a control for some experiments (Figure 1B). Upon bioproduction, RK4-P30-H6 was recovered, in a single step, by immobilized metal affinity chromatography (IMAC). The size and integrity of the construct were confirmed through Western Blot (Figure 1C, left). Notably, we observed that, as predicted, the protein spontaneously organized as monodisperse nanoparticle populations of around 52 nanometers in size, which could be disassembled by submitting the materials to denaturing plus chelating conditions (Figure 1C, right). In contrast, the control GFP-H6, also well-produced (Figure 1D, left), remained unassembled because of the non-cationic character of its N-terminal end (Figure 1D, right).

### 3.2. RK4-p30-H6 Microparticles Promote Consistent and Prolonged Antigen Release

**Figure 2:**
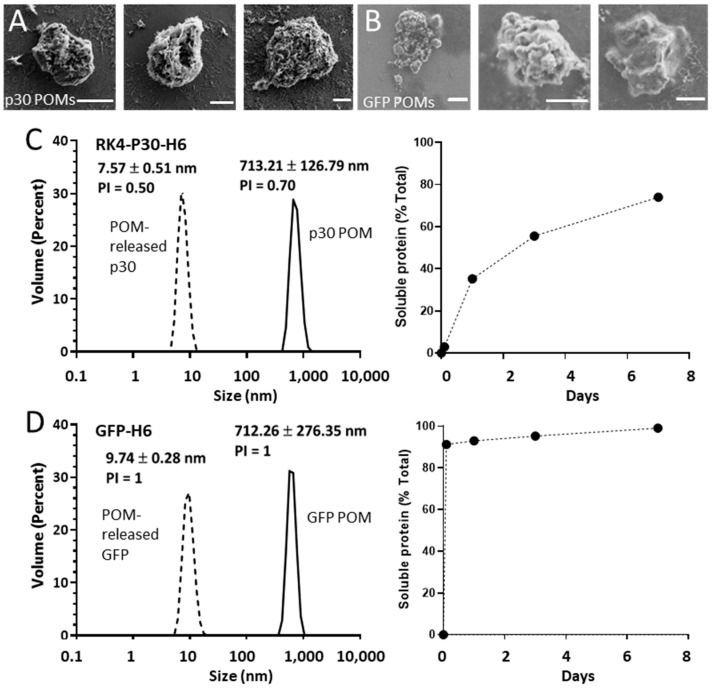
Formation and characterization of p30 POMs and GFP POMs. Representative micrographs of p30 POMs (**A**) and GFP POMs (**B**) obtained by SEM (scale bar represents 1 µm). (**C**) Size of p30 POMs and of the soluble protein released in vitro after seven days as determined by DLS (left). The relative amount of soluble protein released from p30 POMs for seven days is also shown (right). (**D**) Size of GFP POMs and of the soluble protein released from these microparticles as determined by DLS at day seven (left). The relative amount of soluble protein released from GFP POMs for seven days is also depicted (right).

Using pure solutions of RK4-p30-H6 and GFP-H6, we generated secretory microscale granules by mixing the stored pure protein with a zinc chloride solution at a 200:1 divalent cation-to-protein ratio. The formation of these granules occurs through the Zn-mediated cross-molecular clustering via the overhanging H6 tails [10], and it results in mechanically stable particles that leak the forming protein, in vitro and in vivo, during prolonged time periods [12,17]. The size of the resulting materials, named p30- or GFP-POMs (from Protein-Only Microparticles), was determined by both SEM and DLS (Figure 2A,B), resulting in values ranging from 0.5 µm to 4 µm. We also evaluated the protein released from these POMs linked to their slow disintegration, in vitro, under physiological conditions, for seven days. Under this experimental setting, a highly regular and sustained protein leakage was observed during the entire experiment in the case of p30-POMs (Figure 2C), but faster for a large fraction of the GFP-POMs content (Figure 2D). A small fraction of GFP-H6 was, however, released progressively for a few days, in a time-sustained way. The differential leakage pattern of GFP-H6 was probably related to the missing N-terminal cationic peptide, which in this type of modular protein construction shows architectonic roles through electrostatic interactions between protein monomers [13]. The size of the released RK4-p30-H6 and GFP-H6 proteins were compatible with their dimeric forms (Figure 2C,D), that appeared as highly stable. This fact indicated that the building block nanoparticles (sizing around 50 nm) out of which the p30 POMs were formed (Figure 1A) were unstable upon their transit through POMs, disassembling in lower order structures in parallel or immediately after leakage in vitro. In other parallel platforms based on different proteins of oncological interest, the leaked nanoparticles have been shown more stable under in vitro testing [17]. However, we assumed that the material produced here might be more robust in vivo, in which a more crowded ionic environment would be supportive of the intermolecular protein–protein interactions within the nanoparticles [40]. In this context, the nanoparticle version of the antigen might be at least temporarily available for immune stimulation.

### 3.3. Subcutaneous Administration of p30 POMs Are Safe and Immunogenic in Pigs

**Figure 3:**
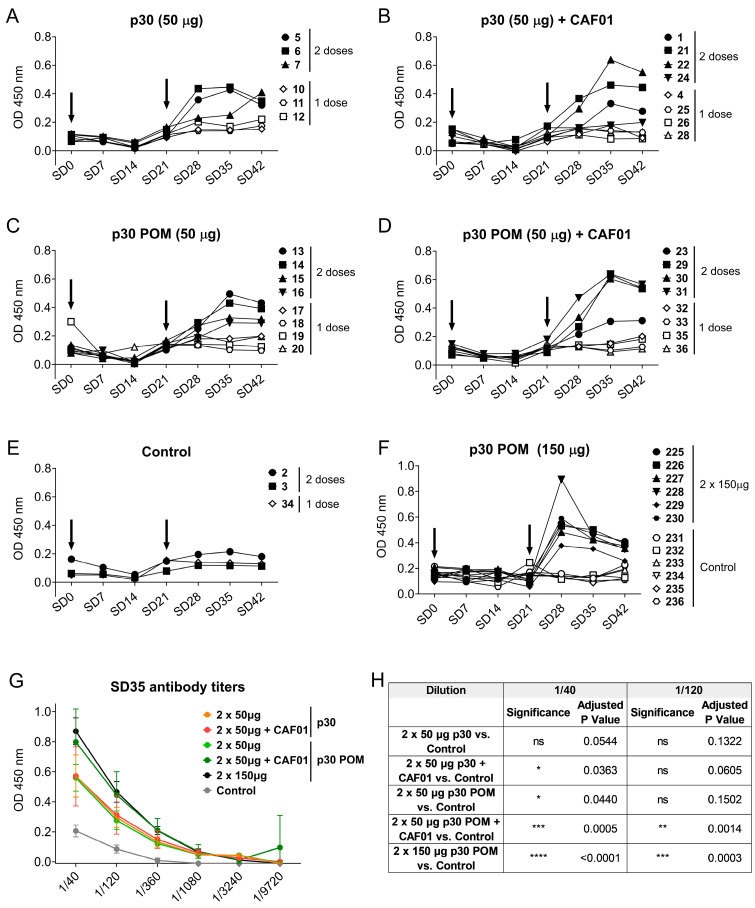
Subcutaneous inoculation of p30 secretory granules in pigs induces ASFV-specific antibodies. ASFV-specific IgGs in sera from pigs inoculated with 50 µg of p30 Soluble (**A**) without or (**B**) with CAF01, or with 50 µg of p30 POMs (**C**) without or (**D**) with CAF01, and (**E**) the control group. (**F**) ASFV-specific IgGs in sera from pigs receiving 150 µg of p30 POMs or PBS as control from the second experiment. Arrows indicate the two vaccination days. (**G**) ASFV-specific IgG titers in pig sera two weeks after the second administration (SD35). (**H**) Statistical analyses of ASFV-specific IgG titers in pig sera two weeks after the second administration (SD35). Statistical significance was determined by one-way ANOVA followed by Tukey’s multiple comparisons test and is displayed in GraphPad style (ns *p* > 0.05, * *p* ≤ 0.05, ** *p* ≤ 0.01, *** *p* ≤ 0.001, **** *p* ≤ 0.0001).

In previous studies, the subcutaneous inoculation of protein-only secretory microparticles formed by therapeutic proteins did not show any side-toxicities while the released protein was fully functional [17]. Here we aimed to assess the safety and immunogenicity of protein-only secretory microparticles for their potential use as slow-release systems for antigens. To do so, the soluble and POMs forms of the ASFV p30 were subcutaneously administered to pigs, and the induced antibody response was analyzed. In each group, pigs received one dose of 50 µg at SD0, and a booster was administered at SD14 in only half of them. For comparative purposes, two additional groups of pigs received the same protein versions but formulated with the commercial adjuvant CAF01. No adverse effects were noticed in the animals after administration of any of the formulations. Neither control pigs injected with PBS nor pigs receiving one single dose of any of the tested protein versions and formulations showed detectable ASFV-specific IgGs at any of the tested time points (Figure 3A–E). In contrast, the administration of two doses of p30 resulted in seroconversion in all cases, detectable from day 7 after the second inoculation, peaking after 14 days, and slowly declining by day 21 (Figure 3A–D). Importantly, the antibody levels induced after two doses of soluble p30 or p30 POM, both in the presence of the adjuvant CAF01, demonstrated the higher capability of the microparticulated protein to enhance the induction of a protein-specific antibody response (Figure 3G,H).

In preceding the testing of secretory granules, doses of 0.5–1 mg were routinely administered to mouse models with a good accumulation of the tumor-targeted released protein in target tissues [17]. Considering the small amount of protein used here (50 µg/dose), we wondered whether increasing the injected POM amount could optimize the antigen formulation, and even trigger a potent immune response that might prevent the booster or the use of adjuvant. To test this hypothesis, six pigs were inoculated twice with 150 µg of p30 POMs, while another group of six control pigs received PBS. Again, no seroconversion was observed after one single shot, but detectable ASFV-specific IgGs were observed after a second administration (Figure 3F). To allow for better comparison between groups, the ASFV-specific IgG titers two weeks after the second administration (SD35) were determined. Remarkably, immunization with soluble p30 alone did not render significant levels compared to the control group, while 50 µg of soluble p30 plus CAF01 showed a slight but significant increase in specific antibodies compared to the control group at a dilution of 1/40 (Figure 3G,H). Notably, the use of p30 POMs without CAF01 also resulted in a significant increase in the antibody levels at this dilution (Figure 3G,H), suggesting that the particulate nature of the antigen was capable of enhancing vaccine immunogenicity without the need of an adjuvant. Indeed, while the inoculation of CAF01with 50 µg of p30 POMs resulted in significantly higher levels of ASFV-specific IgGs at both tested dilutions, 150 µg of p30 POMs without CAF01 rendered even more significantly high ASFV-specific antibody levels (Figure 3G,H).

**Figure 4:**
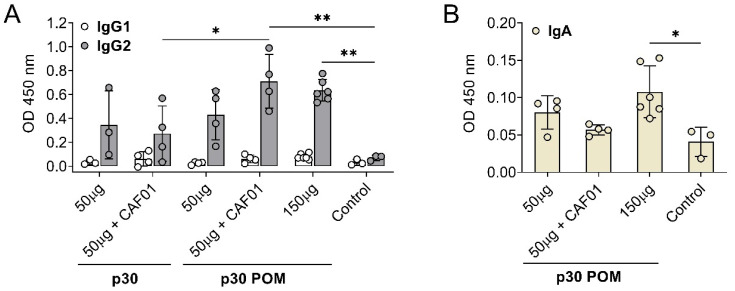
Subcutaneous inoculation of p30 POMs in pigs induces an IgG2 bias and detectable IgA in serum. (**A**) ASFV-specific IgG1 and IgG2 in sera (1/100 dilution) from pigs receiving p30 or p30 POMs two weeks after the second inoculation (SD35) assessed by ELISA. (**B**) ASFV-specific IgA in sera (1/100 dilution) from pigs receiving p30 POMs two weeks after the second inoculation (SD35) determined by ELISA. Statistical significance was determined by one-way ANOVA followed by Tukey’s multiple comparisons test and is displayed in GraphPad style (* *p* ≤ 0.05, ** *p* ≤ 0.01).

To further characterize the antibody response triggered by p30 POMs, the induced IgG isotypes as well as the presence of specific IgA in serum were assessed by ELISA. Immunization with p30, both as the soluble protein version and in the POMs form, induced IgG2 anti-ASFV antibodies but no detectable IgG1 (Figure 4A), indicating a Th1-like bias [41]. This total bias towards a Th1-like response is in contrast with the balanced IgG1/IgG2 profile typically observed in sera from ASF-immune pigs after vaccination with the live attenuated ASFV BA71ΔCD2, a protective vaccine prototype developed by us [41]. Again, only the groups receiving 50 µg of p30 POMs plus F01 or 150 µg of p30 POMs showed statistically significant higher levels of IgG2 compared to the controls, and more uniform levels were found in animals inoculated with the high dose of p30 POMs (Figure 4A).

Notably, significant levels of ASFV-specific IgA were only found in serum after administration of the higher 150 µg p30 POMs amount (Figure 4B), stressing the importance of an adequate dose.

### 3.4. POM Proteins Stimulate the Immune System in a Non-Specific Manner

**Figure 5:**
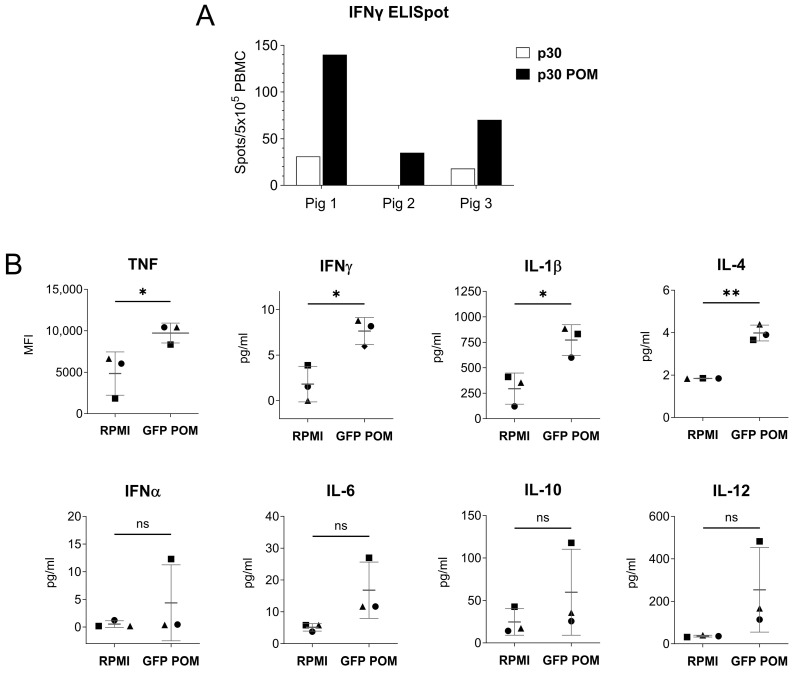
In vitro stimulation with POMs induces non-specific cell activation. (**A**) IFNγ-producing PBMCs from three naive pigs measured by ELISpot assay. Cells were stimulated in vitro with 5 µg/mL of p30 or p30 POMs. (**B**) Cytokine levels in culture supernatants of PAMs stimulated in vitro for 24 h with 5 µg/mL of GFP POMs or left unstimulated (RPMI) quantified by Luminex-based multiplex assay. Statistical significance was determined by unpaired two-tailed t-test for normally distributed data, or two-tailed Mann–Whitney U test for not normally distributed data and is displayed in GraphPad style (*p* > 0.05 ns, * *p* ≤ 0.05, ** *p* ≤ 0.01).

As showed above, the molecular architecture of POMs might have an immunostimulatory capability that overcomes the lack of adjuvant (Figure 4B). This is likely due to the capacity of particulate antigens to enhance vaccine immunogenicity compared to soluble antigens [42]. Thus, to gain further insight on the immunomodulatory properties of POMs, we performed two in vitro experiments to evaluate the capability of POMs to trigger a non-specific stimulation of immune cells. First, PBMCs from three naïve pigs were stimulated with p30 or p30 POMs for 16 h, and IFNγ-producing cells were quantified by ELISpot assay. The results showed that p30 POMs had a higher capability to non-specifically activate PBMCs compared to the non-microparticulated p30 (Figure 5A). Second, pulmonary alveolar macrophages (PAMs) were stimulated in vitro for 24 h with GFP POMs or left untreated (RPMI), and a multiplex Luminex assay was used to assess the levels of cytokines in supernatants. Our results demonstrated that GFP POMs were able to significantly stimulate the secretion of several pro-inflammatory cytokines, including TNF, IFNγ, IL-1β, and IL-4 (Figure 5B). No deleterious effect was observed on PAMs after in vitro stimulation with POMs, supporting their safety profile for future applications.

### 3.5. Subcutaneous Administration of GFP POMs Are Safe and Immunogenic in Mice

**Figure 6:**
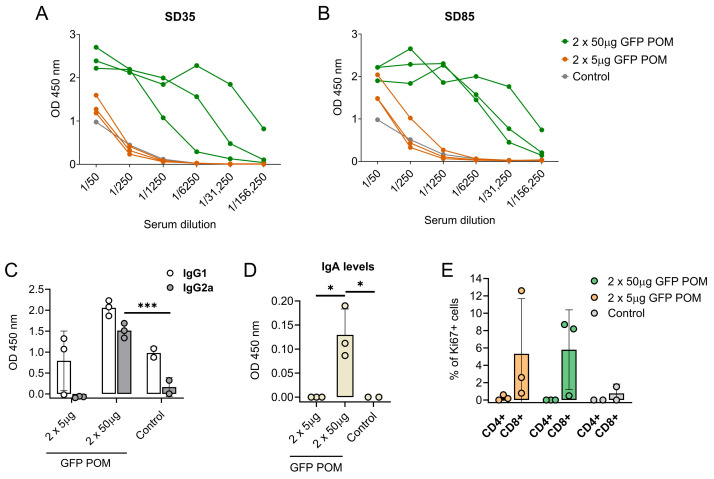
Subcutaneous inoculation of microparticulated GFP induces long-lasting dose-dependent production of GFP-specific antibodies and CD8 T cells in mice. (**A**,**B**) GFP-specific antibody levels in sera from mice receiving GFP POMs assessed by ELISA two (SD35, (**A**)) and nine (SD85, (**B**)) weeks after the second inoculation. (**C**) GFP-specific IgG1 and IgG2a in sera from mice receiving GFP POMs nine weeks after the second inoculation. (**D**) GFP-specific IgA in sera from mice receiving GFP POMs nine weeks after the second inoculation. (**E**) Percentage of proliferating (Ki67+) CD4^+^ and CD8^+^ T cells in splenocytes after in vitro stimulation for five days with 5 µg/mL of GFP POMs. Percentages obtained from untreated cells were subtracted. Statistical significance was assessed by one-way ANOVA followed by Tukey’s multiple comparisons test and is displayed in GraphPad style (* *p* ≤ 0.05, *** *p* ≤ 0.001).

With the intention of compiling more data regarding the immunogenicity and the immune response triggered by POM formulations, our next step was to test them in another animal model, specifically in mice. To do so, BALB/c mice were inoculated twice 2 weeks apart with either 5 or 50 µg of GFP POMs. Administration of GFP POMs in mice induced long-term GFP-specific antibodies in a dose-dependent manner. Thus, only the 50 µg high dose induced significant levels of GFP-specific antibodies that were detectable two weeks after the second shot (SD35, first time point tested) and lasted at least nine weeks (SD85, last time point tested) (Figure 6A,B). The POM formulation of GFP induced significantly higher levels of GFP-specific IgG2 antibodies (Figure 6C), in accordance with what was observed in pigs receiving p30 POMs. Moreover, in this case, levels of IgG2 were also augmented, although not significantly, in mice receiving the high dose of 50 µg of GFP POMs (Figure 6C). Also, in line with what was observed in pigs, anti-GFP IgA was detected in mice serum only when prime boosting with 50 µg of GFP POMs, but not with 5 µg doses (Figure 6D). These results suggested the potential of POM formulations to induce mucosal immunity, as well as the dose dependence of the immune response induced. Two weeks after the second administration, half of the mice were sacrificed to analyze the cellular response induced by GFP POMs. Thus, splenocytes were isolated and stimulated in vitro for 5 days with 5 µg/mL of GFP POMs to be assayed for lymphoproliferative activity by flow cytometry, using the Ki67 proliferation marker. In vitro stimulation of splenocytes obtained from mice immunized with GFP POMs, either with 5 or 50 µg/dose, resulted in the specific proliferation of CD8^+^ T cells (Figure 6E). Despite the proliferation extent not being significantly higher, these data provide the first set of evidence that GFP POMs can induce memory CD8^+^ T cells in mice, even at low concentrations of antigen.

## 4. Discussion

In the context of global health and facing potential pandemic threats, developing new and more efficient immunization systems is an inexcusable need. Much has been learned about vaccination strategies from the still ongoing COVID-19 pandemic, but a consensus has been reached about the fact that both the classical and the recently developed vaccination strategies are still far from optimal [42,43,44,45]. A main and generic problem of the current vaccination boosts is the limited time of exposure to the antigen. In natural infections, the sensing elements of the immune system perceive the immunogens during days or weeks, while pathogen multiplication is active. Among other studies, a recent analysis of the response against the human immunodeficiency virus (HIV) Env protein has determined the relevance of prolonged exposure to relevant antigens in the development of vaccination strategies [46], versus the conventional single antigen shot. In fact, a slow-delivery immunization strategy based on Env over 12 days dramatically improved the immunological outcomes, as such prolonged dosage mimics the features of the immune response to natural infections and expands the durability and effectiveness of such response [46]. Therefore, time-sustained dosage of immunogens is now observed as a ground-breaking and highly promising alternative to the boost-based current approaches [47].

Linked to this scenario, divalent cations such as Zn^2+^ are excellent protein-clustering agents that exploit its interactivity with His residues to crosslink His-tagged polypeptides as nanoparticles or, at higher metal concentrations, nanoparticle-secreting microparticles [10] (Figure 1A). The microparticle version of the resulting materials shows an amyloidal architecture, inner organization, and protein-leaking properties [12]. This is similar to the properties observed in the secretory granules from the mammalian endocrine system that support the secretion of peptide hormones from such granular depots [21,23,48,49]. This category of protein-based dynamic repositories, in the synthetic version described here, has so far been adapted to the release of protein drugs [17]; of course, it might be very convenient for the slow-style approach of antigen release in vaccinology. In addition, compared to other sustained drug delivery systems that require holding through non-functional scaffolding materials [5], bioactive polypeptides, formulated as artificial secretory granules, are self-contained and self-released in the absence of potentially toxic assisting containers. Apart from the time-sustained vaccination ideology [47], the removal of any drug vehicle is also a main goal in the emerging conceptual setting around nanomedical drug delivery [50]. This, of course, would be of perfect applicability to immunization approaches based on selected antigens, under the umbrella of subunit vaccines in contrast to the use of the whole pathogen.

By testing here the above principles with an innovative POM formulation, based on metal-assisted, self-assembling nanoparticles (Figure 1 and Figure 2), we demonstrate the induction of a balanced immune response, including the activation of the innate immunity, as well as antigen-specific antibody and cellular responses (Figure 3, Figure 4, Figure 5 and Figure 6). The activation of the innate immune system by POMs most probably provides an optimal environment for the induction of antigen-specific adaptive immune responses in the moment when the POMs slowly disintegrate in the form of soluble protein components, namely unstable nanoparticles that progressively disassemble, at least in vitro, until their stable dimeric protein forms. Furthermore, the induction of specific IgAs after parenteral immunization in both pig and mice (Figure 4 and Figure 6), opens new expectations for using the secretory granules as a transversal platform to generate broad, systemic, and probably mucosal immunity in any animal species. Of course, further studies are required to test the potential efficacy of POMs in the induction of mucosal immunity upon parenteral or intranasal administration and regarding the potential involvement of traces of bacterial molecules that might be present in the preparations. In this context, the immunogenicity of POMs has to be validated using other antigens, ideally using infection models which allow testing their protective capability. The p30 protein from ASFV has demonstrated to be a good tool to investigate the potential of POMs as a vaccine platform. However, for this particular pathology, this protein should be combined in the future with other ASFV antigens to test its protective capacity against this complex disease [51,52].

In vaccinology, the activation of specific CD8 T cell responses is of high importance to achieve sterilizing immunity against intracellular pathogens. Thus, while antibodies can block the pathogens in fluids, CD8 T cells are the only ones capable to specifically killing the infected cells, also destroying the intracellular pathogen replicating within them. In fact, subunit and inactivated vaccines are traditionally not efficient at inducing CD8+ T cells. The ability of POMs to induce a CD8+ T cell response observed here might be linked to their original, aggregated microscale nature, which can facilitate cross-presentation and, therefore, the priming of CD8 T cell responses [51,52].

Different antibody isotypes play distinct roles in antiviral immunity. Among them, IgG2 antibodies are specifically triggered during Th1-type immune responses [53] and exhibit superior capabilities in activating Fc receptor-mediated effector responses, which are crucial for resolving infections caused by intracellular pathogens. On the other hand, elevated levels of IgG1 are associated with Th2 immune responses and do not stimulate Fc receptor-mediated immune responses as effectively [54]. Both POM formulations tested in this study induced IgG2a of IgG2 antibodies in mice and pigs, respectively. This observation aligns with the activation of CD8^+^ T cells by cross-priming and the induction of a Th1 adaptive response because of the aggregated nature of the microparticles.

## 5. Conclusions

As summarized from the present data, the combination of several intrinsic features of nanoparticle-based POMs, as described here, make them a promising platform for the further development of vaccine candidates. Among them, it is important to stress that (i) the supramolecular architecture of the antigens might be protective from proteolytic degradation in vivo; (ii) the time-extended release of the protein mimics the constant antigen stimulation during a natural infection; (iii) the supramolecular structure of the antigen might act as an adjuvant and promote antigen presentation; and (iv) the delivered antigens might transit from nanoparticles to dimeric forms through intermediate oligomers during the disintegration of the depot. Such a disintegration process might induce specific antibodies and T-helper responses, and also cytotoxic T cells by cross-priming, as already shown for conventional nanoparticle-based vaccines [55]. Together with these features, the easy and cost-effective manufacturing, as well as the safety profile due to their intrinsic molecular purity, make POMs a very promising immunization platform with a high versatility in its further adaptation for new generation vaccine formulation.

## Data Availability

Data will be made available on request.
